# Precision public health: Mapping socioeconomic disparities in opioid dispensations at Swedish pharmacies by Multilevel Analysis of Individual Heterogeneity and Discriminatory Accuracy (MAIHDA)

**DOI:** 10.1371/journal.pone.0220322

**Published:** 2019-08-27

**Authors:** Anna Persmark, Maria Wemrell, Sofia Zettermark, George Leckie, S. V. Subramanian, Juan Merlo

**Affiliations:** 1 Unit for Social Epidemiology, Faculty of Medicine, Lund University, Malmö, Sweden; 2 Department of Gender Studies, Faculty of Social Sciences, Lund University, Lund, Sweden; 3 Centre for Multilevel Modelling, University of Bristol, Bristol, United Kingdom; 4 Department of Social and Behavioral Sciences, Harvard T.H. Chan School of Public Health, Boston, Massachusetts, United States of America; 5 Centre for Primary Health Care Research, Region Skåne, Malmö, Sweden; University of Tennessee Health Science Center, UNITED STATES

## Abstract

**Background:**

In light of the opioid epidemic in the United States, there is growing concern about the use of opioids in Sweden as it may lead to misuse and overuse and, in turn, severe public health problems. However, little is known about the distribution of opioid use across different demographic and socioeconomic dimensions in the Swedish general population. Therefore, we applied an intersectional Multilevel Analysis of Individual Heterogeneity and Discriminatory Accuracy (MAIHDA), to obtain an improved mapping of the risk heterogeneity of and socioeconomic inequalities in opioid prescription receipt.

**Methods and findings:**

Using data from 6,846,106 residents in Sweden aged 18 and above, we constructed 72 intersectional strata from combinations of gender, age, income, cohabitation status, and presence or absence of psychological distress. We modelled the absolute risk (AR) of opioid prescription receipt in a series of multilevel logistic regression models distinguishing between additive and interaction effects. By means of the Variance Partitioning Coefficient (VPC) and the area under the receiver operating characteristic curve (AUC), we quantified the discriminatory accuracy (DA) of the intersectional strata for discerning those who received opioid prescriptions from those who did not.

The AR of opioid prescription receipt ranged from 2.77% (95% CI 2.69–2.86) among low-income men aged 18–34, living alone, without psychological distress, to 28.25% (95% CI 27.95–28.56) among medium-income women aged 65 and older, living alone, with psychological distress. In a model that conflated both additive and interaction effects, the intersectional strata had a fair DA for discerning opioid users from non-users (VPC = 13.2%, AUC = 0.68). However, in the model that decomposed total effects into additive and interaction effects, the VPC was very low (0.42%) indicating the existence of small interaction effects for a number of the intersectional strata.

**Conclusions:**

The intersectional MAIHDA approach aligns with the aims of precision public health, through improving the evidence base for health policy by increasing understanding of both health inequalities and individual heterogeneity. This approach is particularly relevant for socioeconomically conditioned outcomes such as opioid prescription receipt. We have identified intersections of social position within the Swedish population at greater risk for opioid prescription receipt.

## Introduction

### Opioid prescription in Sweden—Reasons to be aware

Opioid prescription and use are rapidly increasing in high income countries [1]. This phenomenon is particularly relevant in the United States, where the existence of an ‘opioid epidemic’ is considered a major threat to public health [2–6]. In Sweden, such an opioid epidemic has not yet been identified [7]. Publicly available information [8] indicates that the percentage of people receiving opioid dispensation from Swedish pharmacies has slightly decreased from 10.2% in 2006 to 9.38% in 2015. Men’s values are lower than those of women, during this period, but present a similar trend. Thus, the proportion of individuals filling a prescription has remained fairly constant. In any case, the current US opioid epidemic and the evidence indicating risks of misuse, overdose and mortality due to opioid prescription [2, 3, 5–7, 9, 10] are concerning facts. These circumstances warrant the investigation of the use of opioids in the Swedish population, even in the absence of an overt epidemic in Sweden [7, 11].

### Factors associated with opioid use in the population

Recent epidemiological analyses indicate that several demographic and socioeconomic factors are associated with opioid use. While lower socioeconomic positions (e.g. lower educational and income levels) appear to correlate with greater risk of opioid prescription receipt [12–15] and greater risk of opioid-related mortality [10], age and gender seem heterogeneously associated with opioid misuse and opioid related mortality [3, 7, 16–18].

For example, in the US (2015), while nonmedical use of opioids appears to be most common among young adults, death due to opioid overdose is most common in adults aged 45–54, while those aged 55–64 have experienced the greatest increase in overdose mortality in the past decade [3], and while one study found similar distribution of opioid prescription among men and women [7], another found higher rates for women [17]. There is further an established correlation between weak social support and substance use and misuse [11, 19, 20]. Alongside demographic and socioeconomic factors, psychological distress appears to be strongly associated with opioid use, dependency and misuse [7, 11, 14, 17].

Knowledge of the demographic and socioeconomic factors that, together with psychological distress, are associated with opioid use is of major relevance for public health. An improved mapping of risk in the population will not only allow for effective, targeted strategies to promote the safe use of opioids, but also enable identification of societal factors that condition opioid use over and above individual needs. However, this phenomenon is complex and not only related to singular demographic or socioeconomic dimensions.

### Towards precision public health by Multilevel Analysis of Individual Heterogeneity and Discriminatory Accuracy (MAIHDA) within an intersectional framework

Research in (social) epidemiology typically applies demographic and socioeconomic dimensions like gender, ethnicity, income, education and occupation to analyses of disparities in health. Generally, we observe worse health outcomes among disadvantaged groups, such as those defined by low income, migrant status, low educational attainment, or unemployment. However, this conventional epidemiological approach shows some weaknesses.

The conventional approach is based on the study of differences between average risks of groups defined by demographic, socioeconomic or ethnic categorizations, but without consideration of the discriminatory accuracy (DA) of such categorizations [21]. While differences between group averages do not account for heterogeneities within or overlaps between groups, measures of DA inform on the ability of a diagnostic tool, statistical model or risk factor to correctly discriminate between people with or without the outcome of interest [22]. In the presence of substantial heterogeneity, DA can be low even if differences in average risk are large. In such cases, reliance on measures of average risk alone may compromise the effectiveness of public health interventions and of individual risk assessments. In addition, potentially stigmatizing assessments of individuals or groups as high-risk, and targeted intervention aimed towards such groups, should be avoided if DA is low [21, 23].

Drawing on previous research stressing the relevance of measuring the DA of categorizations in public health [21, 23–29], we argue that attention to DA aligns with the increasing emphasis on precision medicine, as well as with a corresponding interest in adopting a precision public health perspective [30] as a means to provide “precision prevention” through offering “the right intervention to the right population at the right time” (p 398) [30]. The latter is furthered through more accurate methods for measuring exposures and vulnerabilities, including but not limited to biomedical susceptibilities on the individual level [30]. While precision medicine has been criticized for being overly focused on the individual level, thus disregarding crucial social determinants of health at the population level [31, 32], this tension between individual and population perspectives can be addressed by the adoption of a conceptual multilevel framework, which integrates individual and population levels of analysis. While enabling analysis of both between- and within-population heterogeneity, multilevel analysis does not dislocate these levels from each other, but informs on the share of individual heterogeneity that exists at the population level [21, 33].

Multilevel analysis of individual heterogeneity and discriminatory accuracy (MAIHDA), termed by Merlo [34] and building on previous efforts towards investigating variation between and within contexts [22, 35, 36], has recently been applied in social epidemiology [37–39]. As we discuss elsewhere [22, 23, 34], MAIHDA converges with the current movement toward precision (i.e., individualized, personalized or stratified) medicine, and its efforts toward understanding individual heterogeneity.

A second weakness of conventional (social) epidemiological approaches to the study of health disparities, is that these typically investigate singular demographic and socioeconomic dimensions, like gender, ethnicity, income and education, while failing to account for complexities arising from the intersections of such dimensions. To address this shortcoming, intersectionality theory has increasingly been proposed [40] and used [21, 26, 29, 34, 37, 40–43] as a theoretical and methodological framework in social epidemiology. Famously introduced by the legal scholar Kimberlé Crenshaw in 1989 [44], intersectionality theory proposes that societal power structures or axes of differentiation and oppression such as racism and sexism cannot be fully understood through singular categorical analyses, but must be analyzed as complex, overlapping, and interacting systems [44].

While previously having been used for investigation of geographical [35, 45] and institutional effects [46, 47] on individual outcomes, MAIDHA has recently incorporated an intersectional approach. Based on studies by Jones et al. [48] and Evans et al. [41, 49] as well as by our research group [34, 37, 50], intersectional MAIHDA models the individual health outcome (i.e., opioid prescription receipt in the present case) through a multilevel logistic regression analysis of individuals (level 1) nested within intersectional strata (level 2). The intersectional strata consist of a matrix of all possible combinations, or intersections, of the socioeconomic and demographic variables under study.

### An intersectional approach for precision public health

The intersectional MAIHDA provides a number of technical and substantive benefits for the investigation of socioeconomic disparities in health [34, 38, 39, 48, 49]. Pertinently, the intersectional MAIHDA conceptualizes intersecting dimensions of social position not as essential characteristics of individuals but as contexts, comparable to neighborhoods and subject to political and social change [21, 34, 42, 50]. This is done through the modelling of individuals (level 1, in the multilevel logistic regression analysis) nested within strata consisting of intersecting social positions (level 2, in the analysis) [49]. Thus, the noted tendency in quantitative intersectional study toward assigning causative factors to individuals rather than to structures or processes is avoided [51]. The risk of “blaming the victim” thereby diminishes, as the interpretational focus on differences between strata is directed toward contextual factors. Further, in contrast to conventional approaches, MAIDHA does not require the selection of reference categories (typically male, white, et c.), and thus avoids the associated tendency to reinforce existing norms of primacy (male, white, et c.) [38] while also enabling the analysis of both multiply marginalized strata and strata of mixed marginalization and privilege [38].

Benefits of MAIHDA also include improved scalability, through increased capability to accommodate many intersectional strata, as compared to main effect regression analyses which require geometrical growth of strata through each individual dimension (e.g., gender, race/ethnicity or age) that is added to the matrix. MAIDHA offers improved model parsimony, as intersectional strata are modeled with only one random effects parameter rather than with separate coefficients for each stratum, and it gives an increased reliability of estimates pertaining to small-size groups, through precision-weighted estimates of risk for each intersectional stratum, by means of empirical Bayes, posterior, or shrunken predictions [48]. The intersectional MAIHDA also informs on the possible existence of multiple stratum specific interactions of effects in the additive scale. This represents a step forward for interaction analysis, as the study of interactions in the additive scale has so far been restricted to very few variables at a time [52]. In addition, the intersectional contingency table or matrix itself, produced by intersectional MAIHDA, provides a detailed mapping of the distribution of risk, here of opioid prescription receipt in the population.

Finally, a major reason for using MAIDHA [34] is that the multilevel analysis of variance considers the total individual variance (i.e., the propensity toward opioid prescription receipt) as a continuum that can be decomposed at different levels of the analysis, thus enabling the simultaneous exploration of both between-group and within-group components of individual heterogeneity. Using MAIHDA, group effects are thereby appraised not only through the assessment of differences between strata averages (e.g. relative risk or absolute risk differences), but also through gauging of the share of the individual heterogeneity (i.e., variance in the underlying risk of using opioids) that exists at the group level [22, 33–35, 50]. This is the basis of the concept of intra-class correlation coefficient (ICC) or, in more general terms, of the variance partition coefficient (VPC), which is a standard measure for the assessment of clustering or of general contextual effects in multilevel regression models [34–36, 45, 53–55]. The VPC is thereby a measure of DA, as it discerns the accuracy of the categories under study for classifying individuals with regards to the outcome [21, 23–29, 34, 50]. A corresponding measure can also be obtained using the area under the ROC curve (AUC) [56]. By thus quantifying the DA of the intersectional strata, intersectional MAIHDA enables avoidance of what has been referred to as the “tyranny of the averages” in epidemiology [23–25], i.e., the attribution of the group average risk to all individuals in that group without consideration of the individual heterogeneity of outcomes around that average value. In principle, the larger the VPC, the larger is the share of individual variance attributable to the intersectional strata level. A high VPC corresponds with an *(inter)categorical* [57] intersectional approach, which directs focus toward existing disparities between intersectional strata. Meanwhile, a low VPC relates to an *anti-categorical* [57] approach, which questions the validity or usefulness of the social or intersectional categorizations under study, in relation to the specific outcome at hand [29, 34].

### Aims

In the interest of precision public health, and building on the background presented above, we applied an intersectional MAIHDA to analyze opioid prescription receipt in the total adult population residing in Sweden during 2010–2011. We combined demographic, socioeconomic and health characteristics to construct intersectional strata and thereby obtain improved information on the distribution and socioeconomic determinants of opioid prescription receipt in the Swedish population. Thus, we aim to provide an improved basis for decision making in public health, in line with the aims of precision public health, regarding which population groups need targeting for prevention of opioid prescription receipt. In doing so, we also seek to contribute to the use of intersectionality as a useful and relevant analytical framework within social epidemiology [34].

## Population and methods

### Database

This study is based on the analysis of data from a large record linkage database constructed through the merging of several nation-wide registers, via the unique personal identification number possessed by residents of Sweden. The Swedish Prescribed Drug Register contains information about every drug dispensation made in Sweden (excluding nursing homes and hospital wards) by the Anatomical Therapeutic Chemical (ATC) code. The Swedish Patient Register contains all inpatient and outpatient hospital diagnoses coded according to the 10th International Classification of Diseases (ICD-10) [58, 59]. The registers mentioned above are administered by the Swedish National Board of Health and Welfare. Finally, the Longitudinal Integration Database for Health Insurance and Labor Market Studies, administered by Statistics Sweden, provides demographic and socioeconomic information.

#### Ethics

The record linkage was performed by the Swedish National Board of Health and Welfare (Socialstyrelsen) and Statistics Sweden (Statistiska Centralbyrån) after revision by their data safety committees and approval by the Regional Ethics Review Board in Southern Sweden (Regionala Etikprövningsnämnden i Lund). Personal identifiers were removed before the database was delivered to the research group.

#### Data accessibility

The original databases used in our study are available from the Swedish National Board of Health and Welfare and Statistics Sweden. In Sweden, register data are protected by strict rules of confidentiality [60] but can be made available for research after a special review including approval of the research project by both an Ethics Review Board and the authorities’ own data safety committees. The Swedish authorities under the Ministry of Health and Social Affairs do not provide individual level data to researchers abroad. Instead, they normally advise researchers in other countries to cooperate with Swedish colleagues, to whom they can provide data according to standard legal provisions and procedures. However, for the current study, it is technically possible to perform the analysis using a matrix defined by categories of variables (see the section on intersectional strata). Consequently, the analyses can be performed using the extended table presented in S1 and S2 Tables. This table is fully anonymized and contains a considerable number of individuals in each cell. Therefore, to increase transparency and facilitate the replication of our analysis, we provide the table data as a Stata dataset (see S1 Data) with an accompanying fully annotated Stata Do-file (see S1 Stata Do-file).

#### Study population

The study population consists of all adults (aged 18 and older) residing in Sweden from January 1^st^ to December 31^st^, 2010. We excluded a small number of individuals with missing sociodemographic data (gender, age, income or civil status) and, similarly to Shah, Hayes [61], we also excluded individuals with previous overt pain related diagnoses received at a hospital visit (ICD codes: G43.0-.3, G43.8–43.9, G44.0-G44.4, G44.8, M25.5, M54.0-M54.9, M79.1, M19.6, N80.0–80.9, R07.0-.4, R10.0-.4, R51.9, R52.0-.2, R52.9), or cancer diagnoses (ICD codes: C00-D48), or previous substance use disorders (ICD codes: F10-F19). Our reason for exclusion of individuals with previous pain related diagnoses was our choice to examine the part of the population whose opioid prescription receipt was not directly medically warranted. In other words, our study aims focus toward potentially unwarranted use of opioids in the population, rather than toward treatment of diagnoses for which opioids are medically indicated.

**Fig 1:**
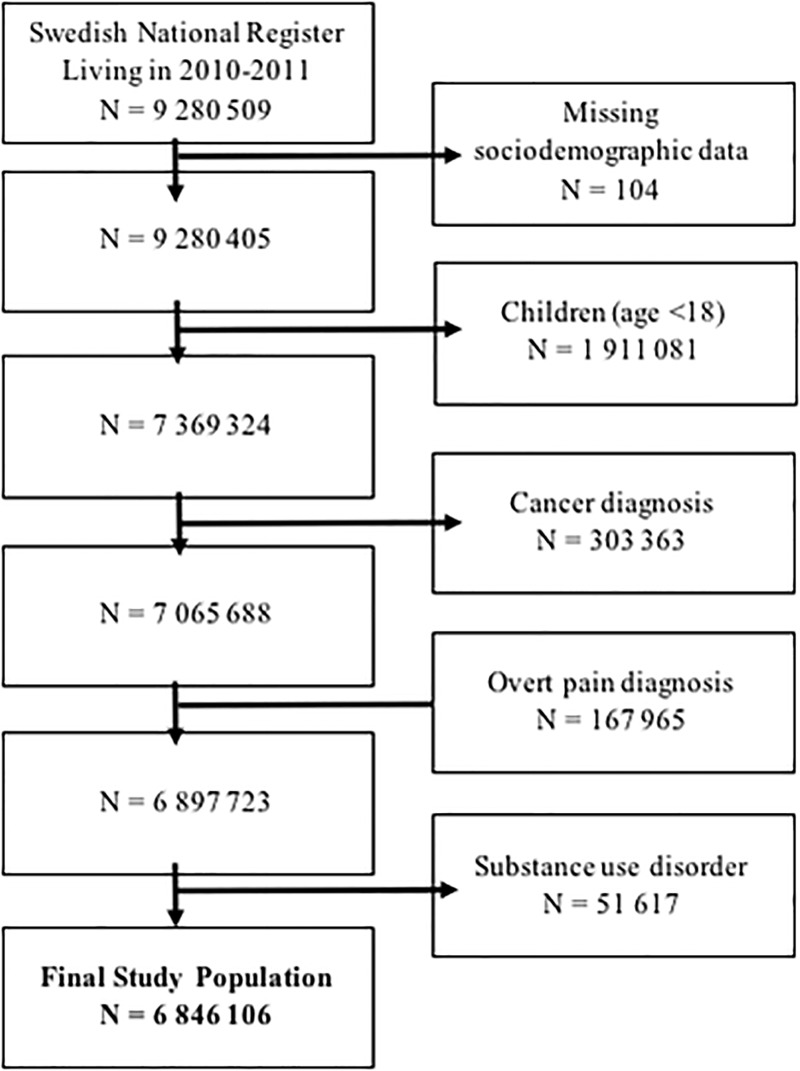
Study population flowchart. We began with all individuals registered in Sweden on December 31, 2011, and excluded children, those with a cancer diagnosis, a pain diagnosis, or a previous substance use disorder diagnosis during 2010, as well as a small number of individuals with missing sociodemographic data.

The final cohort consisted of 6,846,106 adults (Fig 1).

### Assessment of variables

The dichotomous outcome variable was *opioid prescription receipt* (ATC code N02A) between January 1, 2011 and December 31, 2011 (yes vs. no). This broad operationalization includes psychiatric conditions that do not directly warrant opioid prescription, but may still motivate such prescription [62].

*Gender* was a binary variable (men and women). While this binary categorization may be problematic, no other gender identification was available in the data.

We divided *age* into three categories: educational and early working age (18–34 years); later working age (35–64 years); and retirement age and beyond (65+ years).

As a measure of socioeconomic position [63], we use individualized disposable household *income* as provided by Statistics Sweden. To obtain this variable, the total household income after taxes was divided by the number of individuals in the household, and every individual received an age-specific weight. We then categorized these income level into tertiles.

We dichotomized *cohabitation status* as living alone or cohabiting with another adult. Single adults living with children were categorized as living alone.

To operationalize *psychological distress*, we used proxy information on previous dispensation (from January 1, 2010 to December 31, 2010) of neuroleptics and psychoanaleptics (ATC codes N05 and N06 respectively), and on the existence of any diagnosis of a mental or behavioral disorder recorded at a hospital (ICD-10 codes F00 to F99). Codes F10 to F19 were not included this definition as they were exclusion criteria in the study. We defined psychological distress as present when such a dispensation or diagnosis code had been recorded.

By including psychological distress in the intersectional strata, we have strayed somewhat from typical conceptions of intersectionality, as psychological health may not be a social category in and of itself. However, there is evidence that stigmatizing conditions such as mental health disorders have a tangible impact on well-being [64]. Such stigma could have structural effects similar to those of gender, racialization, or class. In addition, exposure to stressful life events and the ability to insulate oneself from them has been conceptualized as one dimension of income as a social determinant of health [38]. Further, mental health disorders have been previously found to be associated with higher rates of opioid use [6, 13, 65–67]. The effects of mental health, both in terms of stigma and the physical implications of psychological discomfort, warrant further investigation in relation to prescription opioid use. This is particularly relevant as while psychological distress is not an indication for opioid prescription, it is known that use of opioids is a maladaptive coping strategy among people suffering from such distress [68].

#### Intersectional strata

We created 72 intersectional strata consisting of all possible combinations of categories of gender, age, income level, cohabitation status, and psychological distress, on the basis of available data and known determinants of opioid prescription receipt (Table 1).

**Table 1:** Absolute risk of opioid prescription receipt: Model 1–3.

Gender		Age			Income			Living alone		Psych. distress		Observed opioid use (%)	Model 1 Simple Intersectional	Model 2b Age Adjusted	Model 2e Psych. Adjusted	Model 3 Interaction Effects
Male	Fem	18–34	35–64	65+	High	Med	Low	No	Yes	No	Yes					
												7.25	-	-	-	REF
												9.95	-	-	-	1.20(1.14,1.27)
												4.38	-	REF	-	REF
												8.51	-	2.00(1.42,2.69)	-	1.97(1.84,2.11)
												14.51	-	2.75(1.98,3.77)	-	2.71(2.53,2.89)
												7.25	-	-	-	REF
												9.38	-	-	-	1.19(1.11,1.27)
												9.47	-	-	-	1.18(1.10,1.26)
												7.95	-	-	-	REF
												9.67	-	-	-	1.09(1.04,1.16)
												6.34	-	-	REF	REF
												19.81	-	-	2.89(2.36,3.52)	2.87(2.71,3.02)
										**Variance (SD)**			0.499(0.087)	0.327(0.057)	0.212(0.037)	0.0138(0.0026)
										**VPC (%)**			13.16	9.04	6.05	0.42
										**PCV (%)**			-	34.4	57.5	97.2

Between-intersectional-strata variance, Variance Partition Coefficient (VPC), Proportional Change in Variance (PCV), and absolute risk for opioid use with 95% credible intervals (CI) for the simple intersectional model 1 (simple components of variance analysis), partially-adjusted model 2 (adjusted for age or for psychological distress) and intersectional interaction model 3 are here shown. See S1 and S2 Tables and S1 Statistical Details for additional information.

### An intersectional MAIHDA

We performed an intersectional MAIHDA [34, 37, 41, 48, 49] with individuals at the first level and the 72 intersectional strata at the second level. The risk of being dispensed a prescription of opioids was thus analysed through three successive multilevel logistic regression models. We estimated the predicted risk and 95% credible interval (CI) associated with each stratum. Technical details of these models (S1 Statistical Details), as well as the Stata dataset (S1 Data) and Do-files (S1 Stata Do-file) which can be used to replicate the analyses, are presented in the supporting information.

#### Model 1: Simple intersectional model

The first model included only an intercept and a random effect for the intersectional strata with no covariates. The purpose of this model was two-fold. First, we performed the simple analysis of components of variance in order to calculate the VPC, which indicates the share of the total individual variance in the propensity for opioid prescription receipt that is accounted for at the intersectional strata level. To calculate the VPC, we used the most popular version derived from the latent response formulation of the model [69, 70], computing it as:

$$VPC=\frac{\sigma_{u}^{2}}{\sigma_{u}^{2}+3.29}$$ (1)

Here, $\sigma_{u}^{2}$ denotes the between-stratum variance in the propensity for opioid prescription receipt while 3.29 indicates the within-stratum between-individual variance constrained equal to the variance of the standard logistic distribution. We multiplied the VPC by 100 and interpreted it as the percentage share of the individual variance which lies between strata.

For additional information, complementary to the VPC, we also calculated the area under the receiver operating characteristic curve (AUC) using the predicted probabilities obtained from each model. The AUC measures the ability of the model to classify individuals with or without the outcome (e.g., presence or absence of opioid prescription receipt) as a function of individuals’ predicted probabilities, thus also measuring DA [56, 71, 72]. The AUC takes a value between 1 and 0.5, where 1 represents perfect discrimination and 0.5 indicates that the covariates have no predictive power [73] (see S1 Statistical Details).

The second purpose of model 1 was to use the shrunken predicted stratum random effects to calculate stratum-specific risks of opioid prescription receipt and, thereby, to obtain an improved mapping of the disparities in opioid prescription receipt. For this purpose, we calculated the risk of opioid prescription receipt and its 95% CI for every intersectional stratum. To do so, and in order to use an additive scale, we transformed the predicted logit (log-odds) of using opioids in in stratum *j* into the predicted probability of opioid use in stratum *j* (see S1 Statistical Details). As the predicted probability in our study represents the absolute risk (AR) of an opioid dispensation, we use the term “risk” rather than predicted probability.

#### Model 2: Partially-adjusted intersectional model

The purpose of the *partially adjusted* model was to quantify the degree to which the different dimensions used to construct the intersectional strata contributed to the between-stratum variance observed in the previous model. In different versions of model 2, we expanded model 1 by adjusting for one covariate at a time (i.e., a different model for each dimension). Thereafter, we calculated the Proportional Change in the between-stratum Variance (PCV):

$$PCV=\frac{\sigma_{u(1)}^{2}-\sigma_{u(2)}^{2}}{\sigma_{u(1)}^{2}}$$ (2)

where $\sigma_{u(1)}^{2}$ and $\sigma_{u(2)}^{2}$ denote the between stratum variance obtained from models 1 and 2 respectively. PCVs are typically multiplied by 100 and reported as percentages.

#### Model 3: Intersectional interaction model

Model 3 expands on model 1 by simultaneously including all of the variables used to construct the intersectional strata as covariates with fixed effect regression coefficients. In this way, model 3 disentangles the main (additive) effects from the interaction effects. In the absence of stratum specific interactions, the main effects of the variables used to construct the intersectional strata (i.e., gender, age, income level, cohabitation status, and psychological distress) would completely explain the between stratum variance and all 72 stratum random effects would equal zero. If this is not the case, and assuming no relevant variables were omitted, the stratum random effects represent the existence of interaction effects between the variables. Therefore, in model 3 the stratum variance and the corresponding VPC inform on the existence of intersectional multiplicative interaction effects, at least in relation to the set of variables included.

We also used model 3 to calculate the total risk of opioid prescription receipt (based on main and interaction effects) and the risk of opioid prescription receipt based on the main effects only. By subtracting the risk attributable to main effects only from the total risk, we isolated the absolute risk due to interaction (ARI) in the additive scale for each intersectional stratum. A positive ARI means that individuals in that intersectional stratum have a *higher* risk than expected based on the simple addition of the risks conveyed by the variables that define the intersectional stratum, while a negative ARI means a *lower* risk than expected. We also calculated 95% CIs for all ARs as well as ARIs.

#### Software

We ran the models using MLwiN 3.00 [74, 75] by calling it from within Stata 14.1 using the *runmlwin* command [76]. The estimations were performed using Markov chain Monte Carlo (MCMC) methods [77].

## Results

**Fig 2:**
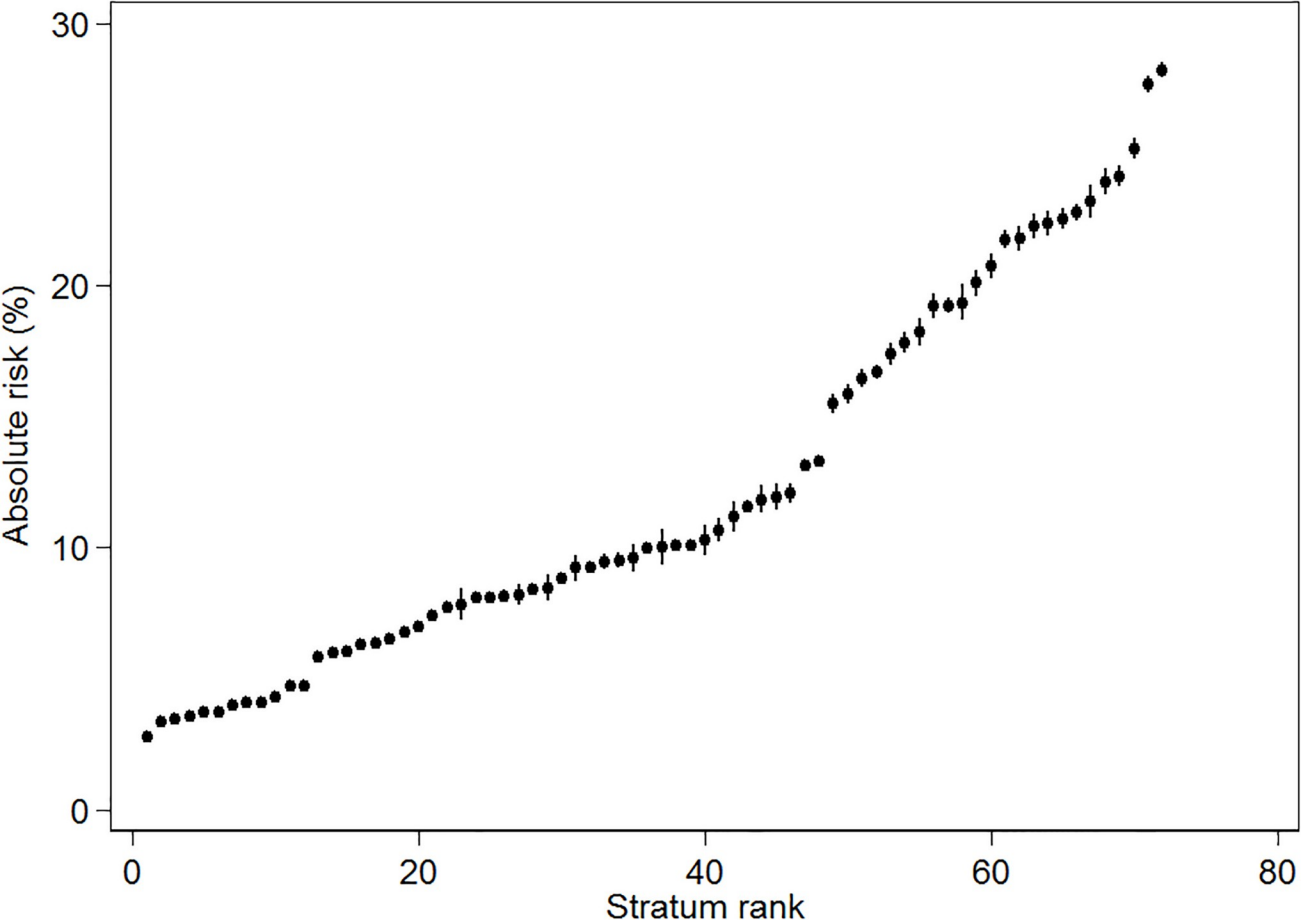
Absolute risk of opioid prescription receipt for intersectional strata. Absolute risk of opioid prescription receipt and 95% Credible Intervals (CI) by intersectional strata for individuals aged 18 years or more residing in Sweden from January 1, 2010 to December 31, 2011. Exact numerical values for each stratum are presented in S1 and S2 Tables.

Fig 2 maps the simple intersectional model (model 1) strata-specific ARs for opioid prescription receipt. The numerical values are available in S1 and S2 Tables. We found the lowest AR for opioid prescription receipt among low income men, aged 18–34, living alone, without psychological distress (AR = 2.77%, 95% CI 2.69–2.86). We observed the highest AR for prescription of opioids in the stratum consisting of medium income women, aged 65-years or older, living alone, with psychological distress (AR = 28.25%, 95% CI 27.95–28.56). That is, the highest AR was 10 times higher than in the group with the lowest AR.

Overall, model 1 (Table 1) shows that the AR for opioid prescription receipt tended to be higher in strata with psychological distress, and lower in strata including younger age.

**Fig 3:**
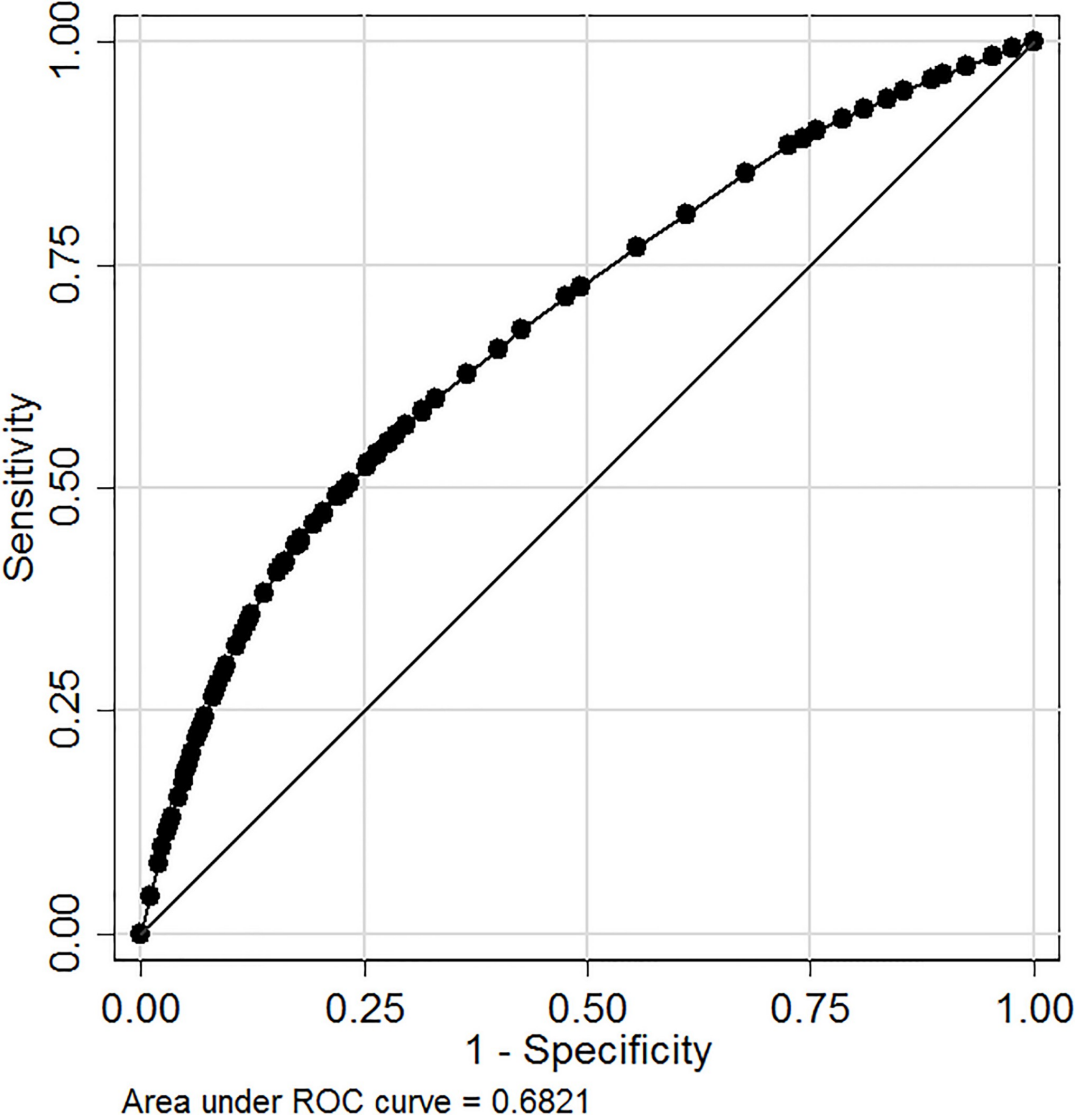
Discriminatory accuracy of intersectional strata. ROC curve analysis obtained in the simple intersectional model 1, quantifying the discriminatory accuracy of the intersectional strata for classifying individuals according to opioid prescription receipt.

The VPC from this simple intersectional model further indicates that as much as 13.2% of the total variance among individuals was located at the intersectional strata level (Table 1). The AUC for model 1 was 0.68 (Fig 3), suggesting a moderate DA.

We can ascertain how much of the between stratum variance that is explained by the components of the intersectional strata by comparing the VPC and PCV values. In model 2 we adjusted for each of the intersectional component variables separately. Age was found to explain 34.4% of the between stratum variance, leading the VPC to drop to 9.04%. Psychological distress explains 57.5% of the between stratum variance, leading the VPC to drop to 6.05%.

**Fig 4:**
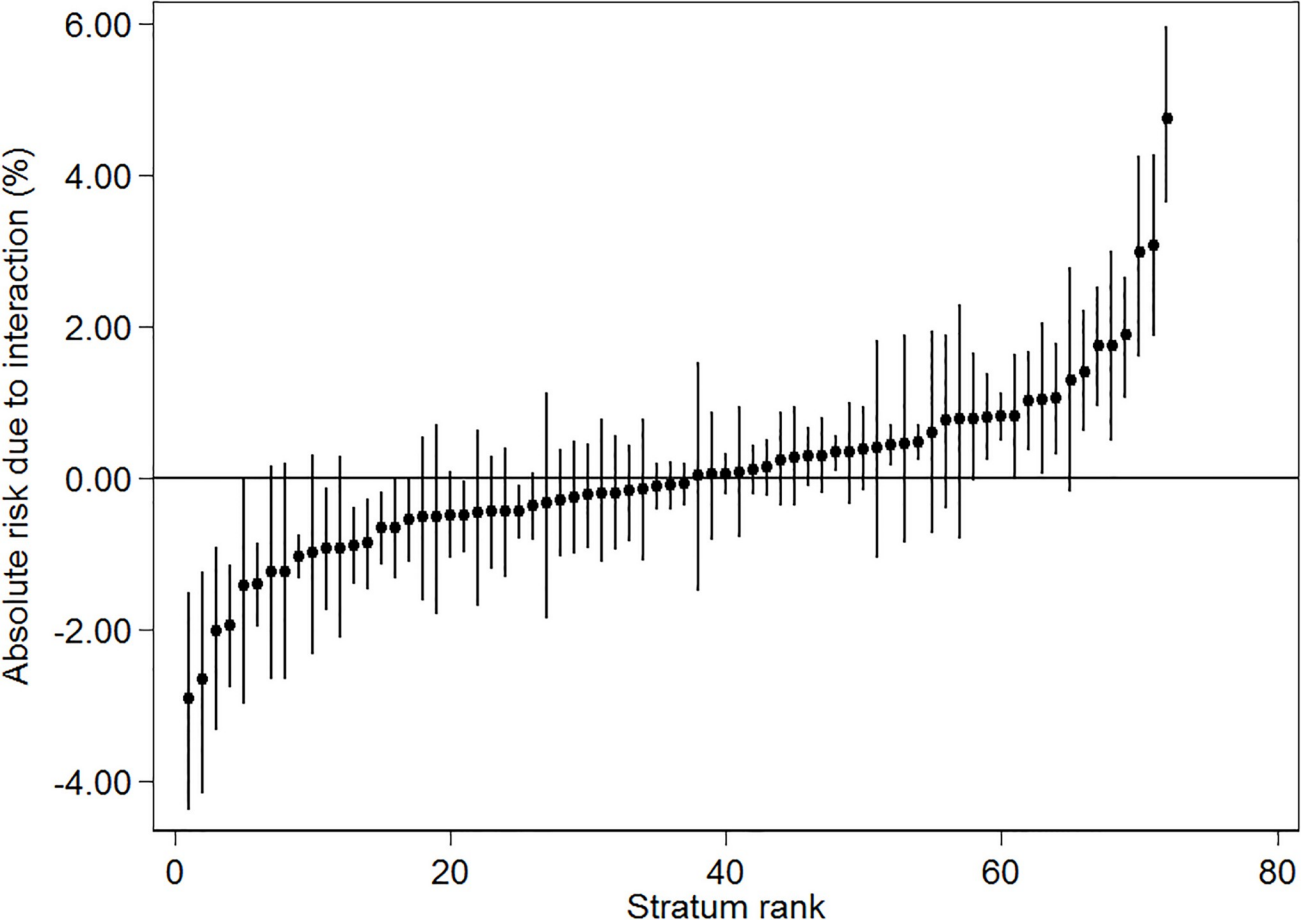
Absolute risk due to interaction. Absolute risk due to interaction (ARI) obtained in the intersectional interaction model 3 in relation to opioid prescription receipt during 2011 for adults residing in Sweden on December 31, 2010, by intersectional strata. Point estimates are ARIs and 95% Credible Intervals (CI). Exact numerical values for each stratum are presented in S1 and S2 Tables.

The intersectional interaction model (model 3) reduced the between-strata variance considerably (PCV = 97.2%) indicating that the differences between strata were mainly due to the additive, rather than the interaction, effects of the variables used for their definition. However, we could still observe conclusive ARIs for certain strata (Fig 4).

Table 2 shows the five most positive and the five most negative ARIs observed in model 3 (see S2 Table for ARI values and 95% CIs for all strata). The largest positive (i.e. hazardous) ARIs were found among the strata comprising men and women with low income and psychological distress, aged 35–64 and cohabiting, and among low income women living alone, aged 35–64 years, with psychological distress.

**Table 2:** Absolute risk due to interaction (ARI), model 3.

Stratum #	Gender		Age			Income			Living alone		Psych. distr.		Model 3 Total Predicted Percentage (95% Credible Interval)		Model 3 Main Effects only Predicted Percentage (95% Credible Interval)		Model 3 Interaction Effects = Total—Main Effects (95% Credible Interval)	
	Male	Fem	18–34	35–64	65+	High	Med	Low	No	Yes	No	Yes						
	*Five Intersectional Strata with the most negative (protective) Interaction Effects*																	
62													19.27	(18.81, 19.74)	22.18	(20.82, 23.56)	-2.91	(-4.38, -1.50)
66													22.59	(22.22, 23.01)	25.24	(23.82, 26.71)	-2.65	(-4.17, -1.23)
52													16.49	(7.87, 8.70)	18.50	(17.43, 19.74)	-2.02	(-3.33, -0.90)
12													8.28	(16.14, 16.83)	10.22	(9.50, 10.96)	-1.94	(-2.76, -1.15)
64													22.33	(6.41, 6.59)	23.75	(22.4, 25.24)	-1.42	(-2.98, 0.01)
	*Five Intersectional Strata with the most positive (hazardous) Interaction Effects*																	
20													20.10	(19.66, 20.61)	18.35	(17.19, 19.55)	1.75	(0.49, 3.00)
71													13.29	(13.09, 13.49)	11.38	(10.63, 12.18)	1.90	(1.06, 2.67)
56													24.17	(23.76, 24.59)	21.19	(19.95, 22.47)	2.98	(1.59, 4.25)
58													22.80	(22.45, 23.16)	19.72	(18.51, 20.92)	3.07	(1.88, 4.28)
22													21.78	(21.32, 22.24)	17.03	(15.87, 18.12)	4.75	(3.63, 5.98)

The table shows the intersectional strata with the five most positive and the five most negative absolute risk due to interaction (ARI) observed in the intersectional interaction model 3. The ARI is calculated by subtracting the absolute risk due to the main effects of the variables that define the intersectional strata from the total stratum specific absolute risk.

The largest negative (i.e. protective) ARIs occurred among women aged 65 and older, cohabiting, with high or medium income and with psychological distress, as well as among high income women living alone, aged 35–64, with psychological distress.

**Fig 5:**
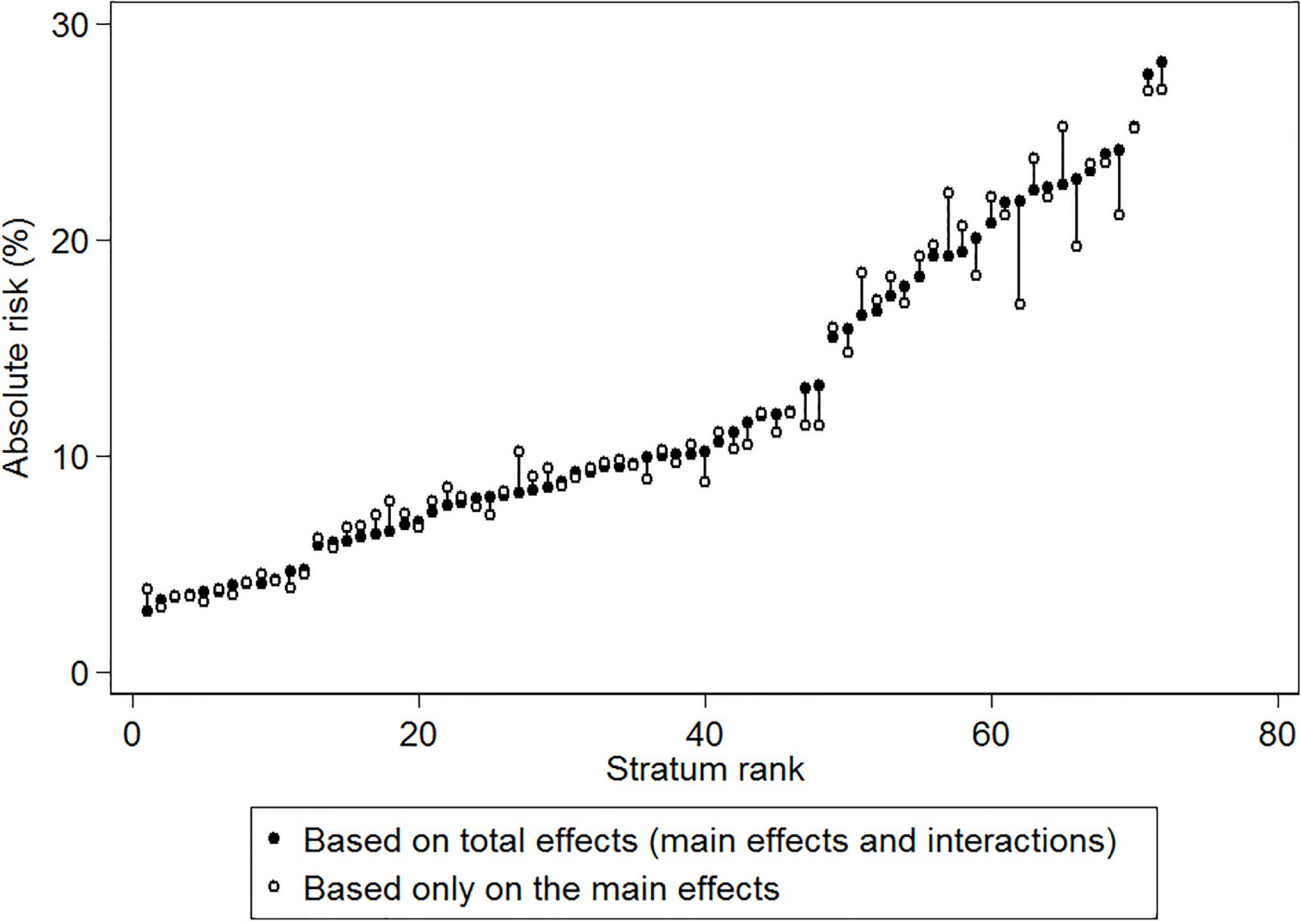
Absolute risk due to interaction: Total and main effects. Presentation of the absolute risk due to interaction (ARI) calculated by subtracting the absolute risk due to the main effects of the variables that define the intersectional strata from the total stratum specific absolute risk. Exact numerical values for each stratum are presented in S2 Table.

We present the differences between ARs due to total stratum specific effects and ARs due to only main effects of the variables that define the intersectional strata in Fig 5.

## Discussion and conclusions

### More precise information on socioeconomic disparities and heterogeneity of risk

Our intersectional MAIHDA study detected socioeconomic disparities in the absolute risk of opioid prescription receipt and, thereby, confirms findings from previous studies both within Sweden [12] and from the United States [13, 14]. However, our findings reveal that these disparities are not unidimensional, but rather intersectional and complex. The stratification of the population in accordance with an intersectional perspective that considers gender, age, income, cohabitation status and, in particular, psychological distress, provides more precise information for identifying candidate population groups for targeted interventions, to promote evidence-based opioid use and to prevent the potential misuse of opioids. By providing measures of DA, we avoid the risk of “tyranny of the averages” and of inaccurate identification of risk groups [23]. The DA of the intersectional stratification used was moderate (as indicated by the VPC and AUC). Therefore, public health policies should not only focus on the intersectional strata with a high absolute risk of opioid prescription receipt, but also on the population as a whole.

In correspondence with other recent studies [41, 42, 49], our analysis applies an innovative method for investigation of socioeconomic disparities in health outcomes in general, and in the use of prescription opioids in particular. As compared to more conventional studies, our intersectional matrix provides a more nuanced understanding of the complex associations between opioid use, psychological distress and relevant socioeconomic factors such as age, gender, and income [1, 3]. The stratum-specific interaction effects we calculated allow us to explore the unique ways in which the intersections influence the risk of opioid prescription receipt. In this way, intersectional MAIHDA contributes toward precision public health aiming to improve targeted prevention strategies based on representative population data [30]. Simple categorizations are misleading, as is evidenced by the strata with hazardous and protective interaction effects. In particular, the strata comprised by cohabiting low income men and women with psychological distress had a greater risk of opioid prescription receipt than would be predicted by the covariate main effects alone. Particular attention should therefore be paid to these population groups when prescribing opioids.

### Limitations

Despite the benefits of applying MAIHDA within an intersectional framework, some limitations should be noted.

First, while MAIHDA is an appropriate method for analyzing strata with low numbers of individuals, because it provides precision weighted estimates [48], as with any other form of analysis the accuracy with which we can estimate the stratum specific absolute risks will increase with sample size. In other words, in spite of the inherent advantage of the shrinkage factor to prevent erratic estimations for small strata, larger samples of individuals are always preferred to ensure the most reliable estimates for all strata. In our study of nearly 7 million individuals, we did not face this particular challenge.

As our intersectional matrix was composed by a limited number of variables, we cannot exclude the possibility that the observed ARIs are not true interactions but are rather due to the main effect of some omitted variable. However, it is possible that omitted variables are mediators of the socioeconomic variables defining the strata, rather than common causes (i.e., confounders) of being located in a specific intersectional stratum and using opioids. In any case, the VPC of 0.4% in the interaction analysis represents the upper bound of the possible interactions.

Although the sociodemographic categories selected to construct the intersectional strata were based on *a priori* assumptions from previous studies, we were limited by the available data (for example, binary gender categories) and we did not have access to prescription data of individuals residing in nursing homes or in-hospital care data. Also, in the Swedish classification of family units, unmarried adults without children are categorized as living alone even if they share a home, and this categorization may thus be overestimated [78]. Furthermore, previous findings in Sweden indicate that low education is associated with increased risk of opioid use [12]. Income and education are both proxies for socioeconomic position and when deciding to include a dimension of socioeconomic position in the definition of the strata we selected income. Even if the multilevel analysis accounts for the reliability of the strata information (i.e., through shrunken residuals), preventing under/overestimation of the strata averages, it does not increase reliability itself. Incorporating more dimensions in the strata would allow for a better understanding of potential intersectional heterogeneity, but decrease the reliability in some strata. Nevertheless, we believe our strata definition provides an improved picture of opioid use across demographic and socioeconomic dimensions in Sweden.

Third, most intersectional MAIHDA studies have an explorative approach. In conventional studies, the theory and *a priori* hypotheses justifying the investigation of the association between simple measures of socioeconomic position (e.g., education, income and occupation) and health-related outcomes are well established [79]. Therefore, most conventional studies of health inequalities are deductive even if the hypotheses are not always explicitly stated. However, in the analysis of an intersectional matrix we do not necessarily have an established hypothesis for each of the intersectional strata. Even so, this approach provides worthy inductive information on socioeconomic differences in health. The new methodology increases our understanding of the dynamics of privilege and disadvantage that drive the production of health disparities, which is not only interesting from an epidemiological point of view, but also from a socioeconomic one.

Further, and as discussed elsewhere [34], the reader should be aware that there are fundamental differences between qualitative analysis within social sciences and quantitative epidemiology when it comes to the application of intersectional frameworks. From a normative perspective, intersectional social categorizations or identities cannot be decomposed and are not easily captured in statistical models [80, 81]. Nevertheless, from the perspective of social epidemiology, the quantitative analysis and decomposition of intersectional strata, as well as the analysis and validation of *categorical* and *anti-categorical* approaches to intersectionality, seem feasible [34]. We believe that tensions between approaches can be negotiated, for purposes of joining forces to denounce unjust health disparities.

Finally, our study population excluded individuals with previous pain-related diagnoses, due to our interest in investigating opioid prescription receipt that was not directly medically warranted, and our outcome variable was quite broad as it included any opioid prescription related to a range of psychiatric conditions. It is possible that the results would be different, should the study have been operationalized differently.

### In conclusion

Opioid use is a complex topic, encompassing growing concerns about increasing misuse and mortality, economic and social costs, and efficacy for pain management [2, 11]. This study is, to our knowledge, the first to implement an intersectional MAIHDA approach regarding opioid prescription receipt in Sweden, and we have found meaningful patterns of opioid prescription receipt across social strata. We have pointed to social strata with higher absolute risk for opioid prescription receipt, and by decomposing the model into additive effects and interaction effects, we have identified which strata had greater risk than anticipated for opioid prescription receipt. Recent public health data in Sweden indicate that prescription of oxycodone in particular has been increasing [7]. In the case of the USA, oxycodone (OxyContin) was largely responsible for the sharp increase in opioid prescriptions during the 1990s [3], and we therefore propose that a future intersectional MAIHDA study should assess oxycodone in particular. Intersectional MAIHDA has the potential to be an important methodological resource for precision public health. From this perspective, intersectional MAIHDA provides an improved mapping of socioeconomic disparities of health outcomes, and a stronger evidentiary foundation for prevention strategies. Concerning opioid prescription receipt in Sweden, our findings suggest the need for public health policy that targets the groups with the highest risk, but simultaneously employs population-level strategies to promote adequate opioid use in the general population.

## Supporting information

S1 Table Predicted probabilities (%), model 1. Predicted probabilities, ranked lowest to highest.
(DOCX)

S2 Table Predicted probabilities (%), model 3. Predicted probabilities, with 95% Credible Intervals. Values are ranked by interaction effects.
(DOCX)

S1 Statistical Details (DOCX)

S1 Data (DTA)

S1 Stata Do-file (TEXTCLIPPING)

## Abbreviations

AR: Absolute Risk
ARI: Absolute Risk due to Interaction
ATC: Anatomical Therapeutic Code
AUC: Area Under the receiver operating characteristic Curve
DA: Discriminatory Accuracy
ICD-10: 10^th^ International Classification of Disease
MAIHDA: Multilevel Analysis of Individual Heterogeneity and Discriminatory Accuracy
PCV: Proportional Change in the between-stratum Variance
VPC: Variance Partitioning Coefficient
