# Correction: Precision public health: Mapping socioeconomic disparities in opioid dispensations at Swedish pharmacies by Multilevel Analysis of Individual Heterogeneity and Discriminatory Accuracy (MAIHDA)

**DOI:** 10.1371/journal.pone.0224008

**Published:** 2019-10-10

**Authors:** Anna Persmark, Maria Wemrell, Sofia Zettermark, George Leckie, S. V. Subramanian, Juan Merlo

The ORCID iDs are missing for the third, fifth, and sixth author. Please see the authors’ respective ORCID iDs here:

- Sofia Zettermark’s ORCID iD is: 0000-0003-3181-8609 (https://orcid.org/0000-0003-3181-8609).
- S. V. Subramanian’s ORCID iD is: 0000-0003-2365-4165 (https://orcid.org/0000-0003-2365-4165).
- Juan Merlo’s ORCID iD is: 0000-0001-8379-9708 (https://orcid.org/0000-0001-8379-9708)
