# Supplementary material for: Precision public health: Mapping socioeconomic disparities in opioid dispensations at Swedish pharmacies by Multilevel Analysis of Individual Heterogeneity and Discriminatory Accuracy (MAIHDA)
Source: PLoS One. 2019 Aug 27;14(8):e0220322. doi: 10.1371/journal.pone.0220322 (PMC6711500; doi:10.1371/journal.pone.0220322)
Supplement: S1 Table — Predicted probabilities, ranked lowest to highest. (DOCX) [file pone.0220322.s001.docx]

# Supporting information: S1 Table

| **Stratum #** | **Gender** | | **Age** | | | **Income** | | | **Living alone** | | **Psych. distress** | | **N** | **% N02** | **Model 1 Predicted Probability** | | |
| --- | --- | --- | --- | --- | --- | --- | --- | --- | --- | --- | --- | --- | --- | --- | --- | --- | --- |
|  | Male | Fem | 18-34 | 35-64 | 65+ | High | Med | Low | No | Yes | No | Yes |  |  | PP (%) | 95% Credible Interval | |
| 11 |  |  |  |  |  |  |  |  |  |  |  |  | 153175 | 2.77 | 2.77 | 2.69 | 2.86 |
| 1 |  |  |  |  |  |  |  |  |  |  |  |  | 138193 | 3.33 | 3.33 | 3.23 | 3.42 |
| 5 |  |  |  |  |  |  |  |  |  |  |  |  | 197663 | 3.44 | 3.44 | 3.36 | 3.52 |
| 9 |  |  |  |  |  |  |  |  |  |  |  |  | 170354 | 3.56 | 3.57 | 3.48 | 3.66 |
| 3 |  |  |  |  |  |  |  |  |  |  |  |  | 202896 | 3.72 | 3.72 | 3.64 | 3.81 |
| 7 |  |  |  |  |  |  |  |  |  |  |  |  | 79253 | 3.71 | 3.72 | 3.59 | 3.86 |
| 37 |  |  |  |  |  |  |  |  |  |  |  |  | 125420 | 3.99 | 3.99 | 3.89 | 4.11 |
| 45 |  |  |  |  |  |  |  |  |  |  |  |  | 167674 | 4.08 | 4.08 | 3.99 | 4.18 |
| 47 |  |  |  |  |  |  |  |  |  |  |  |  | 159501 | 4.10 | 4.10 | 4.01 | 4.20 |
| 41 |  |  |  |  |  |  |  |  |  |  |  |  | 191703 | 4.29 | 4.29 | 4.19 | 4.38 |
| 39 |  |  |  |  |  |  |  |  |  |  |  |  | 108454 | 4.69 | 4.70 | 4.57 | 4.83 |
| 43 |  |  |  |  |  |  |  |  |  |  |  |  | 96415 | 4.69 | 4.70 | 4.55 | 4.83 |
| 15 |  |  |  |  |  |  |  |  |  |  |  |  | 311275 | 5.83 | 5.83 | 5.76 | 5.91 |
| 13 |  |  |  |  |  |  |  |  |  |  |  |  | 449149 | 6.00 | 6.00 | 5.93 | 6.06 |
| 17 |  |  |  |  |  |  |  |  |  |  |  |  | 363340 | 6.04 | 6.04 | 5.96 | 6.11 |
| 49 |  |  |  |  |  |  |  |  |  |  |  |  | 423073 | 6.27 | 6.27 | 6.20 | 6.34 |
| 23 |  |  |  |  |  |  |  |  |  |  |  |  | 107258 | 6.36 | 6.36 | 6.21 | 6.51 |
| 53 |  |  |  |  |  |  |  |  |  |  |  |  | 332480 | 6.50 | 6.50 | 6.42 | 6.58 |
| 51 |  |  |  |  |  |  |  |  |  |  |  |  | 181202 | 6.79 | 6.79 | 6.67 | 6.90 |
| 21 |  |  |  |  |  |  |  |  |  |  |  |  | 237400 | 6.98 | 6.98 | 6.87 | 7.09 |
| 57 |  |  |  |  |  |  |  |  |  |  |  |  | 228367 | 7.40 | 7.40 | 7.29 | 7.51 |
| 59 |  |  |  |  |  |  |  |  |  |  |  |  | 100980 | 7.70 | 7.70 | 7.54 | 7.86 |
| 2 |  |  |  |  |  |  |  |  |  |  |  |  | 7075 | 7.82 | 7.83 | 7.22 | 8.45 |
| 25 |  |  |  |  |  |  |  |  |  |  |  |  | 115424 | 8.06 | 8.06 | 7.89 | 8.22 |
| 19 |  |  |  |  |  |  |  |  |  |  |  |  | 76273 | 8.09 | 8.09 | 7.89 | 8.30 |
| 27 |  |  |  |  |  |  |  |  |  |  |  |  | 49449 | 8.15 | 8.15 | 7.90 | 8.39 |
| 12 |  |  |  |  |  |  |  |  |  |  |  |  | 17152 | 8.19 | 8.18 | 7.80 | 8.60 |
| 61 |  |  |  |  |  |  |  |  |  |  |  |  | 73361 | 8.39 | 8.39 | 8.20 | 8.60 |
| 6 |  |  |  |  |  |  |  |  |  |  |  |  | 11520 | 8.45 | 8.45 | 7.95 | 8.97 |
| 55 |  |  |  |  |  |  |  |  |  |  |  |  | 71174 | 8.83 | 8.83 | 8.62 | 9.02 |
| 10 |  |  |  |  |  |  |  |  |  |  |  |  | 12685 | 9.24 | 9.23 | 8.73 | 9.71 |
| 29 |  |  |  |  |  |  |  |  |  |  |  |  | 125899 | 9.23 | 9.23 | 9.07 | 9.39 |
| 35 |  |  |  |  |  |  |  |  |  |  |  |  | 40105 | 9.47 | 9.47 | 9.18 | 9.74 |
| 63 |  |  |  |  |  |  |  |  |  |  |  |  | 44433 | 9.51 | 9.52 | 9.25 | 9.81 |
| 38 |  |  |  |  |  |  |  |  |  |  |  |  | 10328 | 9.60 | 9.58 | 9.06 | 10.15 |
| 33 |  |  |  |  |  |  |  |  |  |  |  |  | 102561 | 9.97 | 9.97 | 9.79 | 10.15 |
| 8 |  |  |  |  |  |  |  |  |  |  |  |  | 7713 | 10.02 | 10.02 | 9.33 | 10.69 |
| 31 |  |  |  |  |  |  |  |  |  |  |  |  | 74198 | 10.07 | 10.07 | 9.86 | 10.29 |
| 65 |  |  |  |  |  |  |  |  |  |  |  |  | 97615 | 10.09 | 10.09 | 9.90 | 10.29 |
| 4 |  |  |  |  |  |  |  |  |  |  |  |  | 11197 | 10.29 | 10.29 | 9.74 | 10.84 |
| 42 |  |  |  |  |  |  |  |  |  |  |  |  | 17802 | 10.65 | 10.64 | 10.22 | 11.11 |
| 40 |  |  |  |  |  |  |  |  |  |  |  |  | 11871 | 11.17 | 11.16 | 10.59 | 11.75 |
| 69 |  |  |  |  |  |  |  |  |  |  |  |  | 86728 | 11.57 | 11.58 | 11.36 | 11.79 |
| 44 |  |  |  |  |  |  |  |  |  |  |  |  | 14398 | 11.85 | 11.84 | 11.32 | 12.37 |
| 46 |  |  |  |  |  |  |  |  |  |  |  |  | 16563 | 11.94 | 11.94 | 11.44 | 12.46 |
| 48 |  |  |  |  |  |  |  |  |  |  |  |  | 28653 | 12.07 | 12.07 | 11.69 | 12.45 |
| 67 |  |  |  |  |  |  |  |  |  |  |  |  | 109409 | 13.16 | 13.16 | 12.97 | 13.35 |
| 71 |  |  |  |  |  |  |  |  |  |  |  |  | 101022 | 13.30 | 13.30 | 13.10 | 13.52 |
| 16 |  |  |  |  |  |  |  |  |  |  |  |  | 38194 | 15.48 | 15.48 | 15.13 | 15.85 |
| 14 |  |  |  |  |  |  |  |  |  |  |  |  | 41641 | 15.84 | 15.85 | 15.50 | 16.21 |
| 52 |  |  |  |  |  |  |  |  |  |  |  |  | 47789 | 16.45 | 16.46 | 16.12 | 16.81 |
| 50 |  |  |  |  |  |  |  |  |  |  |  |  | 82048 | 16.69 | 16.69 | 16.45 | 16.96 |
| 24 |  |  |  |  |  |  |  |  |  |  |  |  | 30980 | 17.38 | 17.38 | 16.96 | 17.84 |
| 18 |  |  |  |  |  |  |  |  |  |  |  |  | 35395 | 17.84 | 17.84 | 17.44 | 18.25 |
| 26 |  |  |  |  |  |  |  |  |  |  |  |  | 21532 | 18.24 | 18.24 | 17.71 | 18.77 |
| 62 |  |  |  |  |  |  |  |  |  |  |  |  | 27937 | 19.22 | 19.22 | 18.74 | 19.70 |
| 54 |  |  |  |  |  |  |  |  |  |  |  |  | 68425 | 19.24 | 19.23 | 18.95 | 19.54 |
| 28 |  |  |  |  |  |  |  |  |  |  |  |  | 13541 | 19.37 | 19.35 | 18.69 | 20.05 |
| 20 |  |  |  |  |  |  |  |  |  |  |  |  | 25570 | 20.13 | 20.12 | 19.60 | 20.63 |
| 30 |  |  |  |  |  |  |  |  |  |  |  |  | 29268 | 20.76 | 20.75 | 20.30 | 21.22 |
| 60 |  |  |  |  |  |  |  |  |  |  |  |  | 46126 | 21.77 | 21.78 | 21.43 | 22.14 |
| 22 |  |  |  |  |  |  |  |  |  |  |  |  | 30561 | 21.84 | 21.83 | 21.36 | 22.28 |
| 64 |  |  |  |  |  |  |  |  |  |  |  |  | 26463 | 22.30 | 22.29 | 21.82 | 22.76 |
| 34 |  |  |  |  |  |  |  |  |  |  |  |  | 28734 | 22.44 | 22.41 | 21.94 | 22.86 |
| 66 |  |  |  |  |  |  |  |  |  |  |  |  | 42664 | 22.56 | 22.55 | 22.16 | 22.95 |
| 58 |  |  |  |  |  |  |  |  |  |  |  |  | 57530 | 22.83 | 22.82 | 22.48 | 23.15 |
| 36 |  |  |  |  |  |  |  |  |  |  |  |  | 15570 | 23.22 | 23.23 | 22.58 | 23.87 |
| 32 |  |  |  |  |  |  |  |  |  |  |  |  | 27930 | 23.99 | 23.98 | 23.50 | 24.50 |
| 56 |  |  |  |  |  |  |  |  |  |  |  |  | 39056 | 24.20 | 24.19 | 23.80 | 24.59 |
| 70 |  |  |  |  |  |  |  |  |  |  |  |  | 43299 | 25.25 | 25.25 | 24.87 | 25.67 |
| 72 |  |  |  |  |  |  |  |  |  |  |  |  | 78258 | 27.70 | 27.69 | 27.37 | 28.00 |
| 68 |  |  |  |  |  |  |  |  |  |  |  |  | 87762 | 28.26 | 28.25 | 27.95 | 28.56 |
