# Supplementary material for: Precision public health: Mapping socioeconomic disparities in opioid dispensations at Swedish pharmacies by Multilevel Analysis of Individual Heterogeneity and Discriminatory Accuracy (MAIHDA)
Source: PLoS One. 2019 Aug 27;14(8):e0220322. doi: 10.1371/journal.pone.0220322 (PMC6711500; doi:10.1371/journal.pone.0220322)
Supplement: S2 Table — Predicted probabilities, with 95% Credible Intervals. Values are ranked by interaction effects. (DOCX) [file pone.0220322.s002.docx]

# Supporting information: S2 Table

| **Stratum #** | **Gender** | | **Age** | | | **Income** | | | **Living alone** | | **Psych. distress** | | **Model 3 Total Effects** | | | **Model 3 Main Effects only** | | | **Model 3 Interaction Effects** | | |
| --- | --- | --- | --- | --- | --- | --- | --- | --- | --- | --- | --- | --- | --- | --- | --- | --- | --- | --- | --- | --- | --- |
|  | Male | Fem | 18-34 | 35-64 | 65+ | High | Med | Low | No | Yes | No | Yes | **PP (%)** | **95% Credible Interval** | | **PP (%)** | **95% Credible Interval** | | **PP (%)** | **95% Credible Interval** | |
| 62 |  |  |  |  |  |  |  |  |  |  |  |  | 19.27 | 18.83 | 19.74 | 22.18 | 20.82 | 23.56 | -2.91 | -4.38 | -1.50 |
| 66 |  |  |  |  |  |  |  |  |  |  |  |  | 22.59 | 22.22 | 22.96 | 25.24 | 23.82 | 26.71 | -2.65 | -4.17 | -1.23 |
| 52 |  |  |  |  |  |  |  |  |  |  |  |  | 16.49 | 16.14 | 16.82 | 18.5 | 17.43 | 19.74 | -2.02 | -3.33 | -0.90 |
| 12 |  |  |  |  |  |  |  |  |  |  |  |  | 8.28 | 7.87 | 8.67 | 10.22 | 9.50 | 10.96 | -1.94 | -2.76 | -1.15 |
| 64 |  |  |  |  |  |  |  |  |  |  |  |  | 22.33 | 21.83 | 22.85 | 23.75 | 22.4 | 25.24 | -1.42 | -2.98 | 0.01 |
| 53 |  |  |  |  |  |  |  |  |  |  |  |  | 6.50 | 6.42 | 6.59 | 7.90 | 7.34 | 8.48 | -1.4 | -1.97 | -0.85 |
| 28 |  |  |  |  |  |  |  |  |  |  |  |  | 19.41 | 18.76 | 20.11 | 20.65 | 19.41 | 22.06 | -1.24 | -2.66 | 0.17 |
| 30 |  |  |  |  |  |  |  |  |  |  |  |  | 20.77 | 20.28 | 21.24 | 22.01 | 20.6 | 23.35 | -1.24 | -2.66 | 0.20 |
| 11 |  |  |  |  |  |  |  |  |  |  |  |  | 2.78 | 2.70 | 2.86 | 3.82 | 3.54 | 4.11 | -1.04 | -1.33 | -0.75 |
| 26 |  |  |  |  |  |  |  |  |  |  |  |  | 18.26 | 17.75 | 18.74 | 19.24 | 18.09 | 20.44 | -0.97 | -2.33 | 0.3 |
| 6 |  |  |  |  |  |  |  |  |  |  |  |  | 8.52 | 8.04 | 9.01 | 9.45 | 8.79 | 10.11 | -0.93 | -1.75 | -0.13 |
| 24 |  |  |  |  |  |  |  |  |  |  |  |  | 17.40 | 16.98 | 17.81 | 18.32 | 17.2 | 19.48 | -0.92 | -2.11 | 0.29 |
| 23 |  |  |  |  |  |  |  |  |  |  |  |  | 6.37 | 6.23 | 6.52 | 7.26 | 6.77 | 7.77 | -0.88 | -1.41 | -0.38 |
| 59 |  |  |  |  |  |  |  |  |  |  |  |  | 7.71 | 7.55 | 7.87 | 8.56 | 7.99 | 9.16 | -0.86 | -1.47 | -0.27 |
| 17 |  |  |  |  |  |  |  |  |  |  |  |  | 6.04 | 5.96 | 6.11 | 6.69 | 6.20 | 7.16 | -0.65 | -1.15 | -0.17 |
| 61 |  |  |  |  |  |  |  |  |  |  |  |  | 8.40 | 8.19 | 8.60 | 9.04 | 8.40 | 9.69 | -0.65 | -1.32 | -0.01 |
| 51 |  |  |  |  |  |  |  |  |  |  |  |  | 6.80 | 6.68 | 6.92 | 7.34 | 6.82 | 7.88 | -0.54 | -1.10 | 0.00 |
| 50 |  |  |  |  |  |  |  |  |  |  |  |  | 16.70 | 16.45 | 16.94 | 17.20 | 16.23 | 18.27 | -0.50 | -1.62 | 0.55 |
| 54 |  |  |  |  |  |  |  |  |  |  |  |  | 19.25 | 18.97 | 19.56 | 19.75 | 18.61 | 21.07 | -0.50 | -1.81 | 0.71 |
| 49 |  |  |  |  |  |  |  |  |  |  |  |  | 6.27 | 6.19 | 6.35 | 6.76 | 6.30 | 7.24 | -0.49 | -0.99 | -0.03 |
| 57 |  |  |  |  |  |  |  |  |  |  |  |  | 7.40 | 7.30 | 7.51 | 7.89 | 7.32 | 8.46 | -0.49 | -1.06 | 0.09 |
| 16 |  |  |  |  |  |  |  |  |  |  |  |  | 15.49 | 15.11 | 15.85 | 15.95 | 14.95 | 17.07 | -0.46 | -1.70 | 0.63 |
| 42 |  |  |  |  |  |  |  |  |  |  |  |  | 10.66 | 10.24 | 11.16 | 11.1 | 10.34 | 11.92 | -0.44 | -1.32 | 0.40 |
| 65 |  |  |  |  |  |  |  |  |  |  |  |  | 10.09 | 9.90 | 10.29 | 10.53 | 9.83 | 11.28 | -0.44 | -1.20 | 0.29 |
| 47 |  |  |  |  |  |  |  |  |  |  |  |  | 4.10 | 4.00 | 4.20 | 4.54 | 4.21 | 4.89 | -0.43 | -0.79 | -0.10 |
| 15 |  |  |  |  |  |  |  |  |  |  |  |  | 5.83 | 5.76 | 5.92 | 6.21 | 5.78 | 6.68 | -0.37 | -0.82 | 0.07 |
| 36 |  |  |  |  |  |  |  |  |  |  |  |  | 23.21 | 22.59 | 23.82 | 23.54 | 22.16 | 25.01 | -0.32 | -1.85 | 1.13 |
| 63 |  |  |  |  |  |  |  |  |  |  |  |  | 9.51 | 9.23 | 9.78 | 9.80 | 9.17 | 10.52 | -0.29 | -1.03 | 0.39 |
| 2 |  |  |  |  |  |  |  |  |  |  |  |  | 7.84 | 7.26 | 8.46 | 8.10 | 7.52 | 8.66 | -0.25 | -1.01 | 0.49 |
| 35 |  |  |  |  |  |  |  |  |  |  |  |  | 9.47 | 9.20 | 9.75 | 9.69 | 9.03 | 10.39 | -0.22 | -0.92 | 0.45 |
| 8 |  |  |  |  |  |  |  |  |  |  |  |  | 10.04 | 9.38 | 10.67 | 10.24 | 9.50 | 11.01 | -0.20 | -1.11 | 0.77 |
| 10 |  |  |  |  |  |  |  |  |  |  |  |  | 9.24 | 8.81 | 9.71 | 9.44 | 8.78 | 10.1 | -0.19 | -0.95 | 0.57 |
| 27 |  |  |  |  |  |  |  |  |  |  |  |  | 8.15 | 7.91 | 8.41 | 8.32 | 7.74 | 8.93 | -0.17 | -0.83 | 0.43 |
| 44 |  |  |  |  |  |  |  |  |  |  |  |  | 11.86 | 11.35 | 12.38 | 12.01 | 11.17 | 12.86 | -0.15 | -1.09 | 0.77 |
| 7 |  |  |  |  |  |  |  |  |  |  |  |  | 3.72 | 3.59 | 3.85 | 3.82 | 3.54 | 4.12 | -0.10 | -0.41 | 0.2 |
| 45 |  |  |  |  |  |  |  |  |  |  |  |  | 4.08 | 3.99 | 4.17 | 4.17 | 3.86 | 4.50 | -0.08 | -0.42 | 0.23 |
| 5 |  |  |  |  |  |  |  |  |  |  |  |  | 3.44 | 3.36 | 3.52 | 3.51 | 3.25 | 3.80 | -0.07 | -0.36 | 0.21 |
| 70 |  |  |  |  |  |  |  |  |  |  |  |  | 25.24 | 24.86 | 25.66 | 25.21 | 23.78 | 26.64 | 0.03 | -1.48 | 1.53 |
| 9 |  |  |  |  |  |  |  |  |  |  |  |  | 3.56 | 3.48 | 3.64 | 3.51 | 3.23 | 3.78 | 0.06 | -0.22 | 0.33 |
| 38 |  |  |  |  |  |  |  |  |  |  |  |  | 9.59 | 9.05 | 10.15 | 9.54 | 8.89 | 10.21 | 0.06 | -0.82 | 0.87 |
| 48 |  |  |  |  |  |  |  |  |  |  |  |  | 12.07 | 11.70 | 12.46 | 11.99 | 11.17 | 12.81 | 0.08 | -0.79 | 0.94 |
| 41 |  |  |  |  |  |  |  |  |  |  |  |  | 4.29 | 4.20 | 4.38 | 4.17 | 3.87 | 4.51 | 0.11 | -0.23 | 0.43 |
| 43 |  |  |  |  |  |  |  |  |  |  |  |  | 4.69 | 4.55 | 4.83 | 4.54 | 4.2 | 4.91 | 0.15 | -0.23 | 0.50 |
| 55 |  |  |  |  |  |  |  |  |  |  |  |  | 8.82 | 8.62 | 9.02 | 8.57 | 7.97 | 9.18 | 0.25 | -0.37 | 0.87 |
| 29 |  |  |  |  |  |  |  |  |  |  |  |  | 9.23 | 9.08 | 9.40 | 8.96 | 8.31 | 9.57 | 0.27 | -0.36 | 0.95 |
| 13 |  |  |  |  |  |  |  |  |  |  |  |  | 5.99 | 5.92 | 6.06 | 5.71 | 5.33 | 6.1 | 0.28 | -0.11 | 0.67 |
| 21 |  |  |  |  |  |  |  |  |  |  |  |  | 6.97 | 6.87 | 7.08 | 6.68 | 6.19 | 7.17 | 0.29 | -0.21 | 0.8 |
| 1 |  |  |  |  |  |  |  |  |  |  |  |  | 3.32 | 3.23 | 3.43 | 2.98 | 2.76 | 3.21 | 0.34 | 0.09 | 0.57 |
| 31 |  |  |  |  |  |  |  |  |  |  |  |  | 10.06 | 9.85 | 10.29 | 9.71 | 9.08 | 10.40 | 0.35 | -0.35 | 0.99 |
| 25 |  |  |  |  |  |  |  |  |  |  |  |  | 8.05 | 7.90 | 8.22 | 7.67 | 7.13 | 8.21 | 0.39 | -0.17 | 0.94 |
| 32 |  |  |  |  |  |  |  |  |  |  |  |  | 23.97 | 23.51 | 24.47 | 23.57 | 22.19 | 24.99 | 0.40 | -1.06 | 1.82 |
| 37 |  |  |  |  |  |  |  |  |  |  |  |  | 3.98 | 3.87 | 4.09 | 3.55 | 3.29 | 3.83 | 0.44 | 0.16 | 0.71 |
| 34 |  |  |  |  |  |  |  |  |  |  |  |  | 22.43 | 21.95 | 22.92 | 21.98 | 20.53 | 23.29 | 0.46 | -0.86 | 1.89 |
| 3 |  |  |  |  |  |  |  |  |  |  |  |  | 3.72 | 3.63 | 3.79 | 3.25 | 3.01 | 3.49 | 0.47 | 0.23 | 0.72 |
| 60 |  |  |  |  |  |  |  |  |  |  |  |  | 21.77 | 21.40 | 22.15 | 21.17 | 19.89 | 22.45 | 0.61 | -0.73 | 1.95 |
| 18 |  |  |  |  |  |  |  |  |  |  |  |  | 17.82 | 17.43 | 18.23 | 17.06 | 15.97 | 18.15 | 0.77 | -0.40 | 1.89 |
| 72 |  |  |  |  |  |  |  |  |  |  |  |  | 27.70 | 27.36 | 28.00 | 26.92 | 25.47 | 28.49 | 0.78 | -0.80 | 2.29 |
| 40 |  |  |  |  |  |  |  |  |  |  |  |  | 11.12 | 10.59 | 11.69 | 10.33 | 9.57 | 11.08 | 0.79 | -0.04 | 1.65 |
| 19 |  |  |  |  |  |  |  |  |  |  |  |  | 8.08 | 7.88 | 8.27 | 7.27 | 6.76 | 7.77 | 0.81 | 0.24 | 1.39 |
| 39 |  |  |  |  |  |  |  |  |  |  |  |  | 4.68 | 4.56 | 4.80 | 3.86 | 3.58 | 4.17 | 0.82 | 0.50 | 1.12 |
| 46 |  |  |  |  |  |  |  |  |  |  |  |  | 11.90 | 11.42 | 12.37 | 11.09 | 10.35 | 11.85 | 0.82 | -0.02 | 1.65 |
| 33 |  |  |  |  |  |  |  |  |  |  |  |  | 9.96 | 9.77 | 10.15 | 8.94 | 8.31 | 9.59 | 1.02 | 0.36 | 1.68 |
| 14 |  |  |  |  |  |  |  |  |  |  |  |  | 15.84 | 15.48 | 16.20 | 14.79 | 13.89 | 15.74 | 1.05 | 0.06 | 2.05 |
| 69 |  |  |  |  |  |  |  |  |  |  |  |  | 11.57 | 11.36 | 11.77 | 10.52 | 9.79 | 11.26 | 1.05 | 0.30 | 1.79 |
| 68 |  |  |  |  |  |  |  |  |  |  |  |  | 28.25 | 27.96 | 28.56 | 26.95 | 25.54 | 28.46 | 1.3 | -0.18 | 2.79 |
| 4 |  |  |  |  |  |  |  |  |  |  |  |  | 10.18 | 9.65 | 10.74 | 8.78 | 8.16 | 9.45 | 1.4 | 0.62 | 2.21 |
| 67 |  |  |  |  |  |  |  |  |  |  |  |  | 13.14 | 12.94 | 13.35 | 11.4 | 10.64 | 12.17 | 1.74 | 0.94 | 2.53 |
| 20 |  |  |  |  |  |  |  |  |  |  |  |  | 20.10 | 19.66 | 20.61 | 18.35 | 17.19 | 19.55 | 1.75 | 0.49 | 3.00 |
| 71 |  |  |  |  |  |  |  |  |  |  |  |  | 13.28 | 13.09 | 13.49 | 11.38 | 10.63 | 12.18 | 1.9 | 1.06 | 2.67 |
| 56 |  |  |  |  |  |  |  |  |  |  |  |  | 24.17 | 23.76 | 24.59 | 21.19 | 19.95 | 22.47 | 2.98 | 1.59 | 4.25 |
| 58 |  |  |  |  |  |  |  |  |  |  |  |  | 22.80 | 22.45 | 23.16 | 19.72 | 18.51 | 20.92 | 3.07 | 1.88 | 4.28 |
| 22 |  |  |  |  |  |  |  |  |  |  |  |  | 21.78 | 21.32 | 22.24 | 17.03 | 15.87 | 18.12 | 4.75 | 3.63 | 5.98 |
