# Supplementary material for: Precision public health: Mapping socioeconomic disparities in opioid dispensations at Swedish pharmacies by Multilevel Analysis of Individual Heterogeneity and Discriminatory Accuracy (MAIHDA)
Source: PLoS One. 2019 Aug 27;14(8):e0220322. doi: 10.1371/journal.pone.0220322 (PMC6711500; doi:10.1371/journal.pone.0220322)
Supplement: S1 Statistical Details — (DOCX) [file pone.0220322.s003.docx]

# Supporting information: S1 Statistical details

The studied data contain the following variables:

- stratum: stratum identifier ($j$)
- individual: individual identifier (*i*)
- N02_2011: prescription of opioids (1: prescription, 0: no record of opioid prescription) ($y_{ij}$)
- female: dummy variable for gender (1: female, 0: male) ($x_{1j}$)
- workingage, retireage: dummy variables for later working age (35-64) and retirement age (65 and above), the reference category being early working age (18-34), ($x_{2j}$,$x_{3j}$)
- medinc, lowinc: dummy variables for medium and low income, the reference category being high income, ($x_{4j}$,$x_{5j}$)
- alone: dummy variable for living alone (1: yes, 0: no) ($x_{6j}$)
- psychdiag: dummy variable for the prescription of neuroleptics and psychoanaleptics (ATC codes N05 and N06 respectively) or the existence of a diagnosis of a mental or behavioral disorder (ICD-10 codes F00 to F99, excluding F10 to F19 which were excluded from the study) (1: yes, 0: no) ($x_{7j}$)

Where 72 strata have been defined through all possible combinations of gender, age, income, habitation status, and psychological distress ($72=2\times3\times3\times2\times2$).

The data are two-levelled, with 6,848,106 individuals at level-1 in the data hierarchy nested within 72 strata at level-2. While it is convenient to describe the logistic regression models below in terms of the individual-level data, it will prove computationally inefficient to fit models to a dataset of this size. Thus, when we come to fit the models we will first collapse the data from its traditional ‘individual form’ with one observation per individual to its ‘binomial form’ with one observation per stratum. This data manipulation generates no loss of information as the only variable defined at level-1 is the response variable $y_{ij}$ and all of the information contained in this binary variable can be encapsulated in two new variables storing the total number of individuals and the number of opiate users in each stratum (the ‘denominator’ and the ‘numerator’). We can then fit binomial form versions of our logistic regression models to stratum-level binomial counts or proportions (i.e., the denominator divided by the numerator). The results will be identical to those produced by the traditional individual form versions of the models described below.

**Model 1**

The two-level variance-components logistic regression model for $y_{ij}$ can be written as

$$y_{ij}\sim\mathrm{Binomial}\left( 1,\pi_{j} \right)$$

$$\mathrm{logit} \left( \pi_{j} \right)\equiv\log\left( \frac{\pi_{j}}{1-\pi_{j}} \right)=\beta_{0}+u_{j}$$

$$u_{j}\sim N\left( 0,\sigma_{u}^{2} \right)$$

where $\pi_{j}$ denotes the probability of a prescription for opioids for individuals in stratum $j$, $\beta_{0}$ denotes the intercept, and $u_{j}$ denotes the stratum random effect. The model includes no covariates, so the stratum random effect captures both the main effects of the variables used to define the strata and their two-way and higher order interactions. The stratum random effects are assumed normally distributed with mean zero and constant between-stratum variance $\sigma_{u}^{2}$, a parameter to be estimated.

The latent response formulation of the above model can be written as

$$y_{ij}=\left\{ \begin{aligned} 1\mathrm{if}y_{ij}^{*}\geq0 \\ 0\mathrm{otherwise} \end{aligned} \right.$$

$$y_{ij}^{*}=\beta_{0}+u_{j}+e_{ij}$$

$$u_{j}\sim N\left( 0,\sigma_{u}^{2} \right)$$

$$e_{ij}\sim\Lambda\left( 0,3.29 \right)$$

where $y_{ij}^{*}$ denotes the continuous latent response or propensity of receiving a prescription for opioids and $e_{ij}$ is the individual level residual assumed to follow the standard logistic distribution with mean zero and variance 3.29 ($={\pi^{2}}/3$ where $\pi$ is the mathematical constant $3.141\ldots$).

The degree of clustering in the latent responses is typically summarized by one of two statistics: the Intraclass Correlation Coefficient (ICC) or the Variance Partition Coefficient (VPC). In the current and subsequent models, the formulas for the ICC and VPC coincide.

$$\mathrm{ICC}\equiv\mathrm{VPC}=\frac{\sigma_{u}^{2}}{\sigma_{u}^{2}+3.29}$$

The ICC is interpreted as the expected correlation between the latent responses of two individuals from the same stratum. The VPC is interpreted as the proportion of the latent response variation which lies between strata. The proportion of the latent response variation which lies within strata is given by 1-VPC. These stratum and individual level VPCs are typically multiplied by 100 and reported as percentages.

The probability of having a prescription for opioids in stratum *j* is calculated as

$$\pi_{j}=\mathrm{logit}^{-1} \left( \beta_{0}+u_{j} \right)$$

We constructed receiver operating characteristic curves (ROC) using the predicted probabilities obtained from the different models. The area under the ROC (AUC) [1, 2] is constructed by plotting the true positive fraction (TPF) (i.e., sensitivity) against the false positive fraction (FPF) (i.e., 1 − specificity) for different binary classification thresholds of the predicted probabilities. The AUC measures the ability of the model to classify individuals with or without the outcome (e.g., using or not opioids) as a function of individuals’ predicted probabilities. The AUC takes a value between 1 and 0.5 where 1 represents perfect discrimination while 0.5 suggest the same degree of informativity as that of flipping a coin (i.e., the covariates have no predictive power). The AUC of the model quantifies the accuracy of using stratum membership alone for identifying individuals with the outcome.

**Model 2**

Model 2 extends model 1 by entering one covariate at a time. For example, the model which enters female, the dummy variable for gender, can be written as:

$$\mathrm{logit} \left( \pi_{j} \right)=\beta_{0}+\beta_{1}x_{1j}+u_{j}$$

Adding stratum level covariates to the model will explain the between-stratum variance. The degree to which the between-stratum variance reduces as we move from model 1 to 2 can be expressed by the proportional change in variance (PCV) statistic, calculated as

$$\mathrm{PCV}= \frac{\sigma_{u(1)}^{2}-\sigma_{u(2)}^{2}}{\sigma_{u(1)}^{2}}$$

where $\sigma_{u(1)}^{2}$ and $\sigma_{u(2)}^{2}$ denote the between-stratum variance from models 1 and 2 respectively. PCVs are typically multiplied by 100 and reported as percentages.

The ICC and VPCs for this model are calculated as before, but are now interpreted as conditional ICC and VPCs that summarize the degree of clustering in the adjusted latent responses.

**Model 3**

Model 3 extends models 1 and 2 by entering all of the covariates: female, workingage, retireage, medinc, lowinc, alone, and psychdiag. The model can be written as

$$\mathrm{logit} \left( \pi_{j} \right)=\beta_{0}+\beta_{1}x_{1j}+\beta_{2}x_{2j}+\beta_{3}x_{3j}+\beta_{4}x_{4j}+\beta_{5}x_{5j}+\beta_{6}x_{6j}+\beta_{7}x_{7j}+u_{j}$$

Here the seven fixed effect covariates capture the main effects of the explanatory variables on the log-odds of opioid prescription, while the two-way and all higher order interaction effects between these variables are captured by the random stratum effect $u_{j}$.

Model 3 therefore decomposes the log-odds of an opioid prescription into two parts: the part due to the main effects of the explanatory variables (the fixed-part of the model) and the part due to interactions between the explanatory variables (the random-part of the model: the random stratum effect). Thus, the stratum effect $u_{j}$ quantifies the difference between the log-odds in stratum $j$ when we acknowledge the interaction effects and the log-odds in stratum $j$ when we ignore the interaction effects.

To aid interpretation, we can work on the probability scale. The probability of having a prescription for opioids in stratum $j$ is calculated as

$$\pi_{j}=\mathrm{logit}^{-1} \left( \beta_{0}+\beta_{1}x_{1j}+\beta_{2}x_{2j}+\beta_{3}x_{3j}+\beta_{4}x_{4j}+\beta_{5}x_{5j}+\beta_{6}x_{6j}+\beta_{7}x_{7j}+u_{j} \right)$$

This probability can be decomposed into the part due to the main effects of the explanatory variables $\pi_{j}^{A}$ and the part due to the interactions between the explanatory variables $\pi_{j}^{B}$

$$\pi_{j}=\pi_{j}^{A}+\pi_{j}^{B}$$

We calculate the former as

$$\pi_{j}^{A}=\mathrm{logit}^{-1} \left( \beta_{0}+\beta_{1}x_{1j}+\beta_{2}x_{2j}+\beta_{3}x_{3j}+\beta_{4}x_{4j}+\beta_{5}x_{5j}+\beta_{6}x_{6j}+\beta_{7}x_{7j} \right)$$

We calculate the latter by subtraction.

## **Software**

We ran the models using MLwiN 3.00 [3] by calling it from within Stata 14.1 using the *runmlwin* command [4]. We performed the estimations using Markov chain Monte Carlo (MCMC) methods [5]. We specified diffuse (vague, flat, or minimally informative) prior distributions for all parameters. We used quasilikelihood methods to provide starting values for all parameters. For each model, we specified a burn-in length of 5,000 iterations and a monitoring chain length of 10,000 iterations. Visual assessments of the parameter chains and standard MCMC convergence diagnostics suggested that the lengths of these periods were adequate.

## **References**

1. Wagner P., Merlo J. (2013). Measures of discriminatory accuracy in multilevel analysis. European Journal of Epidemiology, 28 (1, Supplement):135.

2. Merlo J, Wagner P, Ghith N, Leckie G. (2016). An Original Stepwise Multilevel Logistic Regression Analysis of Discriminatory Accuracy: The Case of Neighbourhoods and Health. Plos One, 11(4). doi: ARTN e0153778

3. Charlton, C., Rasbash, J., Browne, W.J., Healy, M. and Cameron, B. (2017). MLwiN Version 3.00. Centre for Multilevel Modelling, University of Bristol.

4. Leckie, G., & Charlton, C. (2013). Runmlwin: A program to run the MLwiN multilevel modelling software from within Stata. Journal of Statistical Software, 52, 1-40.

5. Browne, W.J. (2017). MCMC Estimation in MLwiN v3.00. Centre for Multilevel Modelling, University of Bristol.
